# Genome Investigations of Vector Competence in *Aedes aegypti* to Inform Novel Arbovirus Disease Control Approaches

**DOI:** 10.3390/insects7040058

**Published:** 2016-10-30

**Authors:** David W. Severson, Susanta K. Behura

**Affiliations:** 1 Department of Biological Sciences and Eck Institute for Global Health, University of Notre Dame, Notre Dame, IN 46556, USA; 2 Division of Animal Sciences, University of Missouri, Columbia, MO 65211, USA; behuras@missouri.edu

**Keywords:** *Aedes aegypti*, vector competence, mosquito arbovirus interaction, dengue virus, transcriptome, functional genomics, integrative approach, innate immunity, genetic control

## Abstract

Dengue (DENV), yellow fever, chikungunya, and Zika virus transmission to humans by a mosquito host is confounded by both intrinsic and extrinsic variables. Besides virulence factors of the individual arboviruses, likelihood of virus transmission is subject to variability in the genome of the primary mosquito vector, *Aedes aegypti*. The “vectorial capacity” of *A. aegypti* varies depending upon its density, biting rate, and survival rate, as well as its intrinsic ability to acquire, host and transmit a given arbovirus. This intrinsic ability is known as “vector competence”. Based on whole transcriptome analysis, several genes and pathways have been predicated to have an association with a susceptible or refractory response in *A. aegypti* to DENV infection. However, the functional genomics of vector competence of *A. aegypti* is not well understood, primarily due to lack of integrative approaches in genomic or transcriptomic studies. In this review, we focus on the present status of genomics studies of DENV vector competence in *A. aegypti* as limited information is available relative to the other arboviruses. We propose future areas of research needed to facilitate the integration of vector and virus genomics and environmental factors to work towards better understanding of vector competence and vectorial capacity in natural conditions.

## 1. Introduction

The mosquito *Aedes aegypti* is the principal global vector that transmits dengue (DENV), yellow fever, chikungunya (CHIKV), and Zika virus to humans and is well adapted to urban environments. Dengue virus infection causes dengue fever and can lead to more severe symptoms such as hemorrhagic fever, plasma leakage, and organ impairments in humans. Evidence suggests that ~4 billion people in 128 countries are at risk of dengue infection [1]. Indeed, as many as 390 million cases of dengue likely occur each year globally, with many of these being unapparent [2]. Adding to dengue disease complexity, DENV exists as four common serotypes that circulate globally across most tropical and sub-tropical areas. Despite the long availability of an effective vaccine for yellow fever, epidemics with considerable human mortality continue to occur [3]. Re-emerging arboviruses like CHIKV represent persistent cyclical threats of epidemics [4], while emerging arboviruses like Zika will continue to cause massive outbreaks until sufficient herd immunity develops and will then remain cyclical threats [5]. At present, no effective vaccines are available or imminent for DENV, CHIKV, or Zika, and no drug therapies exist [4,5,6,7]. As a result, mosquito control remains the primary control strategy for prevention of dengue and other arboviruses, yet for multiple reasons, including inevitable emergence of insecticide resistant populations [8,9], it is often ineffective. Novel genetic control strategies are being pursued and developed that target *A. aegypti* [10,11,12], with some practical evidence for associated reductions in mosquito populations [13,14] and/or DENV transmission [15], although these remain to be validated in large-scale field trials. In this review, we focus on the current genomic information available for *A. aegypti* interactions with DENV, as little such information is available relative to other arboviruses.

## 2. Gene by Environment Interactions Determine Vector Competence

The susceptibility of *A. aegypti* to DENV and other arbovirus infection is determined by both intrinsic and extrinsic factors [16]. Adult females are exposed to DENV upon blood feeding on viremic humans, followed by virion infection of the mosquito’s midgut epithelium. Thereafter, the virus must successfully replicate and disseminate to other tissues, especially the salivary glands, in order to subsequently be transmitted to a naïve human host [17]. The likelihood for success or failure in establishment of a DENV infection in the midgut is one of the most important intrinsic factors that define vector competence of the mosquito host. The intrinsic ability of an arthropod vector to either host or defend against virus infection is generally referred to as “vector competence” [18]. Interpreting the association between *A. aegypti* and DENV is a dynamic process that is likely confounded by the inherent variability of the individual genotypes in both mosquito and virus, wherein infection outcome reflects genotype-by-genotype (G_M_xG_V_) interactions [19,20,21]. A wealth of information exists documenting genetic variability in DENV vector competence in *A. aegypti* populations both within and between geographic locations [22,23,24,25,26,27,28,29]. In addition, variability in vector competence to different DENV isolates both within the same serotype and across serotypes has been reported within single *A. aegypti* populations [30,31,32,33,34,35]. Further complicating this relationship, other biotic and abiotic factors are known to interact with and influence phenotypic variance in DENV vector competence [36,37,38]. Thus the association between *A. aegypti* and DENV in natural populations is a highly dynamic process that also involves interactions between mosquito (G_M_) and virus (G_V_) genotypes and prevailing environmental (E) factors (G_M_xG_V_xE). Vectorial capacity (VC) represents the average number of potentially infective bites a vertebrate host will receive per day among all vectors that feed on them [39]. Epidemiologically, VC can be estimated using the following equation derived after work by Ross and Macdonald, as reviewed elsewhere [40,41]:

$$VC=\frac{mbp^{n}a^{2}}{-\ln\;p}$$

where *m* = density of vectors (per host), *p* = survival rate of the vector (per day), *a* = biting rate, *n* = extrinsic incubation period, and *b* = vector competence.

Still, our practical knowledge of the phenotypic outcome of genes due to environmental interactions and the overall impact on vectorial capacity in any arthropod vector/pathogen system remains limited. Factors that influence larval development time can affect the size of adult females, which is considered to be an important aspect of vector competence, vectorial capacity, and overall vector biology including longevity, biting persistence, and blood meal frequency [42,43,44,45]. Further, the aquatic environment encountered by eggs and larvae may have an effect on the repertoire of the microbiome during development, and it is known that the microbiome does regulate mosquito development [46], and eventually egg production in adult females [47]. Nasci [43] demonstrated that the average size of host-seeking *A. aegypti* females is significantly larger than the average size of the emerging population of females, and suggested that the likelihood of surviving to a second gonotrophic cycle increases as adult body size increases. However, small adult mosquitoes are known to probe more often and to take multiple blood meals during a gonotrophic cycle [48], suggesting that small mosquitoes may be more likely to encounter an infective human during their lifetime and could potentially be a more epidemiologically relevant vector. Phenotypic outcome in *A. aegypti* adults is extremely plastic and reflects the interaction of genetic potential with environmental conditions encountered by developing larvae [49]. In general, the inorganic and organic nutrient contents of typical *A. aegypti* breeding sites are low as they are dependent on allochthonous inputs [50], and thus larval nutrition can be a limiting factor. Temperature can also have a major impact on disease transmission by influencing body size, development time, and container productivity [51,52,53].

## 3. Quantitative Genetics of Vector Competence

### 3.1. Innate Immune Response Defines Vector Competence

The innate immune system is broadly conserved across insects and mammals [54,55,56,57,58,59]. It has been well documented that an innate immune response is activated in mosquitoes following infection by a diverse array of pathogens [60,61,62,63,64,65,66]. However, this response has been most often shown to limit pathogenesis in the mosquito host, but does not necessarily prevent them from becoming competent vectors for subsequent pathogen transmission to a human host. Variability in vector competence is largely conditioned by the joint action of a small number of genes [67,68]. Such traits are generally referred to as multigene or quantitative traits, and the individual gene locations as quantitative trait loci (QTL). These QTL define the primary upstream loci directing the vector host response to infection by a pathogen and play key roles in conditioning a susceptible or refractory outcome in the host.

### 3.2. Primary Conditioners of Vector Competence Identified as Quantitative Trait Loci

**Figure 1:**
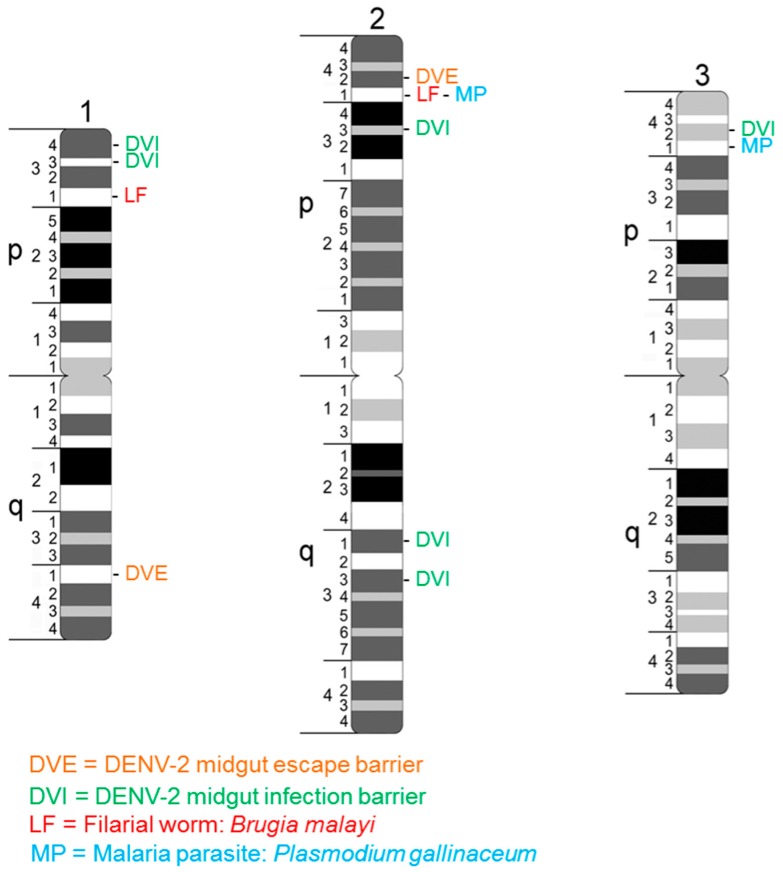
Physical chromosome locations of anchor markers for QTL conditioning vector competence to DENV-2, the protozoan parasite *Plasmodium gallinaceum*, and the metazoan parasite *Brugia malayi*. Adapted from [77].

Our current knowledge on the quantitative genetics of the innate immune response of mosquitoes to pathogen infection largely reflects a collection of diverse and independent studies that have employed various tools/strategies to investigate the fundamental basis for vector competence. Investigations of the quantitative genetics of DENV vector competence in *A. aegypti* females determined that the innate immune response can trigger molecular events that either prohibit the virus from successfully establishing an infection in their midgut epithelium (e.g., a midgut infection barrier, MIB) or following successful midgut epithelium infection and replication somehow prevents virus escape and dissemination to other tissues, particularly the salivary glands (e.g., a midgut escape barrier, MEB] [69]. Subsequent QTL mapping studies in *A. aegypti* have confirmed a multigene mode of DENV vector competence and defined genome regions containing multiple independent QTL [70,71,72]. In addition, QTL studies on other pathogens, including the filarial worm parasite, *Brugia malayi*, and the malaria parasite, *Plasmodium gallinaceum*, identified multiple QTL [73,74,75,76]. Of note, these QTL tend to cluster in only five specific genome regions, irrespective of the pathogen or genetic background of the mosquito host [77], suggesting that these regions (Figure 1) may contain key mutations in loci that ultimately determine vector competence. These loci could represent gene regulatory factors or clusters of genes that are associated with the *A. aegypti* innate immune system. Physical clustering of such genes may be constrained by selective forces. A good example of this can be derived from results of the hundreds of QTL studies performed in maize, where a synthesis of 50 independent QTL studies that examined disease resistance to multiple pathogens across diverse genetic backgrounds demonstrated that certain QTL tended to cluster in specific genome regions across a diverse array of pathogens [78]; the conclusion was that a relatively small number of loci are active in conditioning a susceptible versus resistant innate immune response to all pathogens in maize.

## 4. *A. aegypti* and DENV Interaction Post-Blood Feeding on Infected Human Host

**Figure 2:**
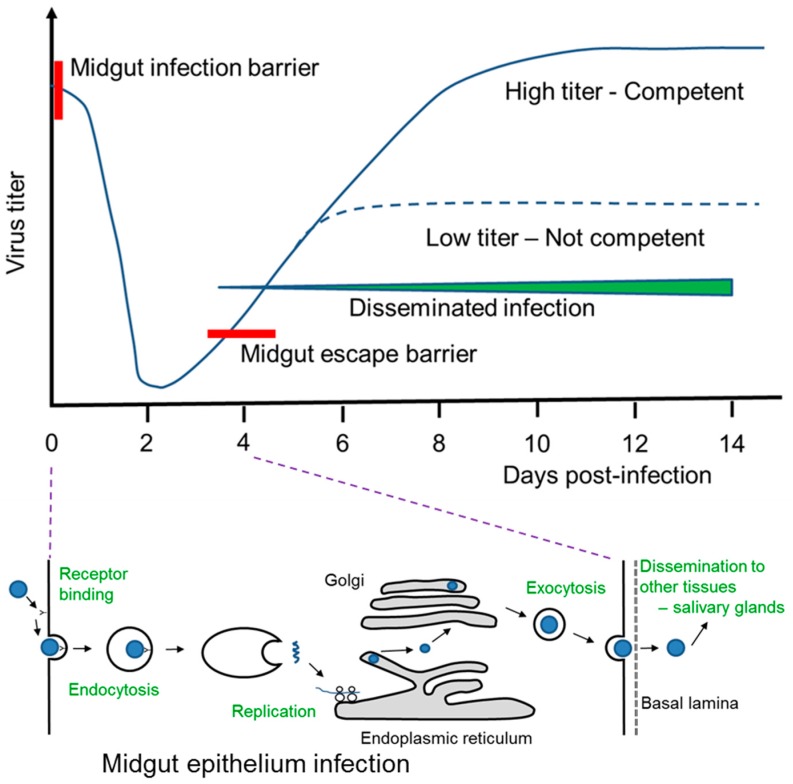
DENV infection and replication cycle in *A. aegypti*. Compiled from [69,79,83,84].

In a genetically susceptible *A. aegypti* female a cascade of events occurs following acquisition of a DENV-infected blood meal from a human host that can ultimately result in infection of the salivary glands and transmission of the virus to naïve human hosts during subsequent blood feeding (Figure 2). The DENV life cycle in mosquito cells is generally homologous to that in human cells, which is well characterized [79]. Initially, the virus binds to surface receptors on midgut epithelia cells [80], followed by clathrin-mediated endocytosis and subsequent endosome maturation, which includes a decrease in pH that promotes viral envelope fusion with the endosome, thereby releasing the viral positive strand RNA genome for replication and translation [81,82]. This process is followed by virus assembly, maturation, and eventually exocytosis from the cell and dissemination to and infection of other cells.

Following ingestion of an infected blood meal, the time required for a susceptible mosquito host to be competent for oral transmission of the virus to another human host is defined as the extrinsic incubation period (EIP) [67]. The length of the EIP is dependent on multiple factors, including genotypes of both mosquito and virus, as well as environmental factors such as the prevailing temperature and the larval rearing conditions. Following ingestion, virus titer drops considerably over the next ~24–48 h (termed as the “eclipse” period) as endocytosis and replication are initiated in the midgut epithelia (Figure 2) [83]. This time period is likely the most critical for determining whether a given mosquito will eventually become competent to transmit virus. That is, for example, in *Aedes albopictus* C6/36 cells, DENV has been shown to successfully bind to the cell membrane and endocytose within 5–7 min following infection, and to colocalize with low pH vesicles like lysosomes by 30 min post-infection [82]. Following replication in a susceptible host, exocytosis occurs and the virus is able to disseminate to and replicate in other tissues, most importantly the salivary glands. The EIP is typically considered to be ~7–14 days, but has been shown to take only ~4 days with some mosquito/virus interactions [84]. Multiple tissues have been linked with barriers to arbovirus infection in mosquitoes [17] that not only include direct cellular activities but also physical barriers such as the basal lamina [84,85]. Of note, at this point no direct evidence exists for a salivary gland barrier to DENV infection in *A. aegypti*, so the prevailing conclusion is that if the virus successfully infects the midgut epithelium and escapes into the hemocoel, that mosquito will become competent for transmission. Thus, the events occurring during the first 3–4 days post-infected blood meal are critical to determining whether a given female becomes competent to transmit virus in a future blood meal. However, evidence does exist indicating that successful virus replication in the midgut and subsequent dissemination to other tissues including the salivary glands and saliva is dependent to some extent on virus titer in the initial blood meal [35,86,87,88]. Thus, virus dissemination to low titers, as evidenced by assessment of leg or head tissues, may not result in successful dissemination to salivary glands and saliva.

## 5. Functional Genomics of Innate Immune Response to DENV

The availability of the *A. aegypti* genome sequence [89] has greatly facilitated efforts to perform broad-scale transcriptome assays of the innate immune response to DENV infection (Table 1). These studies encompass a range of mosquito genetic backgrounds as well as the complete range of the viral EIP in the mosquito host, including multiple tissues. Many of these studies involved mosquito stocks known to be highly susceptible to infection by DENV [90,91,92,93,94], although several have focused on transcriptome comparisons of known susceptible and refractory mosquito stocks [95,96,97,98] as well as a diverse panel of mosquito stocks shown to reflect the full range of susceptibility as determined by midgut virus titers at 7 days post infected blood feeding [99]. Of note, all of these studies have been performed with the same two DENV-2 isolates, New Guinea C and JAM 1409.

The highly conserved nature of systemic innate immune responses of *A. aegypti* and other mosquitoes to various arboviruses including DENV has been reviewed extensively elsewhere and includes the classical immune signaling pathways as well as RNA interference (RNAi) and apoptosis [63,65,66,100,101,102]. Indeed, a consensus across *A. aegypti* transcriptome studies in Table 1 is that, independent of genetic background or post-infection sampling point or tissue in *A. aegypti* females, the innate immune system mounts a vigorous response to DENV infection that is commonly reflected by upregulation of both the Toll and JAK/STAT signaling pathways. Of note, however, the upregulation of these signaling pathways as well as RNAi and apoptotic pathways may be critical to limiting virus titers in mosquito cells; for example, suppression of the RNAi pathway in *A. aegypti* females exposed to Sindbis virus resulted in significant increases in virus titers and decreases in mosquito survival [103]. Thus, demonstration of the upregulation of components of the innate immune system does not in itself explain the basis for genetic refractoriness or susceptibility in a given individual. These findings likely reflect the evolution of mechanisms by DENV and other arboviruses to limit or even manipulate the innate immune system in *A. aegypti* to facilitate its own survival and replication in mosquito cells in a similar manner, as has been well described for DENV interaction with human cells [104]. Indeed, the concept of tolerance of infection vs. resistance to infection by a pathogen has been well documented [105,106]. Additionally, recent evidence suggests that DENV also likely induces autophagy in *A. aegypti* cells to facilitate infection [107] as has again been well documented in human cells [104,108]. To date, however, the key loci that determine a refractory vs. susceptible outcome have not been identified.

## 6. Genome Coevolution and Vector Competence

Arbovirus interactions with both the vertebrate and mosquito host have been shown to have association with coevolutionary processes that influence virus infection mechanisms and the immune systems in both hosts [109,110,111]. Using binomial logistic regression analyses to investigate known DENV responsive genes [96], we found that several intrinsic features such as gene context, intron presence, codon usage bias, paralogy, and derived versus ancestral origin of *A. aegypti* genes each show significant marginal effects with the observed transcriptional responses to DENV infection [112]. Among these features, genes with high codon usage bias were associated primarily with non-responsiveness to DENV infection, while intron-less genes and genes in *A. aegypti* with no ortholog in either *Culex quinquefasciatus* or *Anopheles gambiae* showed a greater association with responsiveness to DENV infection. These findings are consistent with the knowledge that, in general, housekeeping genes evolve slowly and show low intron frequency and high codon usage bias [112,113,114], while genes associated with innate immunity evolve quickly [59,115]. Indeed, the concept of a molecular “arms race” whereby selection by arboviruses may be driving genome evolution in the invertebrate host and vice versa has considerable support [116]. Under this scenario, selection for *A. aegypti* polymorphisms promoting resistance to DENV infection would be countered by matching virus polymorphisms to evade or suppress that response before fixation of resistance alleles in a mosquito population. This outcome also seems well supported by investigations of genotype-by-genotype interactions as they influence the observed *A. aegypti* vector competence at the local or regional levels [19,30,31,117], wherein a reciprocal selective response would reflect variability due to the prevailing environmental conditions. Indeed, we compared *cis*-regulatory element DNA sequences that control gene expression in *A. aegypti* between DENV susceptible and refractory strains and identified >3300 strain-specific single nucleotide polymorphisms within these elements [118].

Further, as DENV is dependent on the host translational apparatus, we and others have reported that DENV isolates show significant biases in synonymous codon usage that are consistent with their geographic origin and likely result from adaptive interaction with the mosquito and human hosts [109,119,120]. We also observed that codon context (the propensity of adjacent codons to consistently pair with themselves or another codon) among Asian and American DENV isolates showed a bias toward (A)(A/T)(A)-(A)(A/T)(A) coding sequences and general avoidance of (C/G)(C/A)(C/G)-(C/G)(C/A)(C/G) coding sequences across all four serotypes [119]. In addition to DENV, we also compared [121] the codon context bias of other flaviviruses, including West Nile virus (WNV) and yellow fever virus (YFV), and determined that codon context bias varies in a bicluster manner with *A. aegypti* genes that have been shown to be differentially expressed following infection by these viruses [92]. This result suggests that codon context sequences of *A. aegypti* and the flaviviruses may play an important role in determining successful infection by these viruses. A recent study demonstrated that host infection ability of DENV can be manipulated by appropriately altering codon context sequences in the virus genome so that it grows efficiently in insect cells but not in mammal cells [122], an approach that holds promise in developing novel attenuated viral vaccine candidates against dengue. Furthermore, DENV has been shown to undergo a bottleneck effect in genetic heterogeneity by alternative cycling in human and mosquito cells [123], thus further supporting the notion of a molecular arms race between mosquito host and virus.

## 7. Conclusions

Our inadequate ability to prevent or control DENV and most other vector-borne diseases demands that we pursue new paradigms. Considerable discussion and research effort is being directed toward applying transgenesis technology to arthropod-borne disease control, with an emphasis on population suppression or replacement [11,124,125,126,127,128,129]. A major focus is to use these techniques to generate mosquitoes that carry genes to disrupt their ability to transmit pathogens. The general concept would be to replace natural mosquito populations with individuals carrying a transgene construct. The most commonly discussed candidate genes for transformation would directly interfere with pathogen development in the mosquito, although genes that would minimize or prevent mosquito/pathogen contact would also be applicable. Transmission blocking vaccines, wherein mosquito antigens provide the basis for inducing pathogen inhibitory antibodies in humans, would prevent the infection and development of the pathogen in the mosquito and thereby interrupt disease transmission [128]. However, it is also well recognized that successful implementation of such strategies will require intensive pre-release and post-release investigations of mosquito ecology and population biology [130]. One important caveat regarding published studies of *A. aegypti* transcriptome responses to DENV and other arboviruses to date is that all have been conducted under optimum lab conditions; we and others [131] would argue that future studies should include similar investigations of the impact on the innate immune response of mosquito and DENV genotypes under typical environmental conditions. Efforts directed at detailed comparative transcriptome studies targeting CHIKV and Zika should be a research priority. This information has the potential to inform and enhance predictive models for vector/virus interaction and disease transmission. Integrative approaches could incorporate information gained on the molecular biology of the innate immune response with traditional aspects of basic mosquito biology such as body size, longevity, and larval density, along with environmental factors, to more accurately predict vector competence at the population level.

## Figures and Tables

**Table 1:** Broad-scale transcriptome assays of the innate immune response of *Aedes aegypti* to dengue virus infection.

Study	*Aedes aegypti* strain(s)	Strain Susceptibility	Dengue Strain ^3^	Mosquito Infection	Sample Point(s) Post-Infection and Tissues	Transcriptome Assay
[90]	Rockefeller	Susceptible ^1^	A	oral	10 days, midgut	Agilent microarrays
					10 days, carcass	
[91]	Rockefeller	Susceptible	A	oral	3 days, 7 days, whole body	Agilent microarrays
[95]	Cali	Susceptible	A	oral	48 h, midgut	Suppressive subtractive hybridization
	Cali	Refractory				
[92]	Rockefeller	Susceptible	A	injection	1 day, 2 days, 7 days, whole body	NimbleGen
[96]	Moyo-R	Refractory	B	oral	3 h, 18 h, whole body	NimbleGen
	Moyo-S					
[98]	Moyo-D	Refractory	B	oral	1 h, 4 h, 1 day, 2 days, 4 days, midgut	Custom cDNA microarrays
	Moyo-S					
[93]	Chetumal	Susceptible	B	oral	1 day, 4 days, midgut	Illumina, RNA-Seq
					14 days, salivary glands	
					1 day, 4 days, 14 days, carcass	
[94]	Rockefeller	Susceptible	A	oral	14 days, salivary glands	Agilent microarrays
					14 days, carcass	
[99]	Rockefeller	High ^2^	A	oral	7 days, midgut	Agilent microarrays
	Orlando	Low			7 days, carcass	
	Waco	Low				
	Puerto Rico	Intermediate				
	Saint Kitts	Intermediate				
	Por Fin	Intermediate				
	Puerto Triunfo	High				
	Singapore	High				
	Bangkok	Low				
[97]	D2S3	Susceptible	B	oral	3 h, 3 days, midgut	Custom cDNA microarrays
	Moyo-D	Refractory				

^1^ Susceptible vs. refractory status based on dissemination from the midgut; ^2^ High, intermediate, low status based on midgut titers relative to Rockefeller strain; ^3^ A = DENV-2 New Guinea C, B = DENV-2 JAM 1409.

## Abbreviations

The following abbreviations are used in this manuscript:

DENV	Dengue virus
G_M_	Genotype of mosquito
G_V_	Genotype of virus
E	Environment
VC	Vectorial capacity
QTL	Quantitative trait locus
MIB	Midgut infection barrier
MEB	Midgut escape barrier
EIP	Extrinsic incubation period
RNAi	RNA interference
WNV	West Nile virus
YF	Yellow fever virus
